# Rabbit Feces as Feed for Ruminants and as an Energy Source

**DOI:** 10.3390/ani4040755

**Published:** 2014-12-05

**Authors:** Pier Giorgio Peiretti, Sonia Tassone, Francesco Gai, Laura Gasco, Giorgio Masoero

**Affiliations:** 1 Institute of Sciences of Food Production, National Research Council, Largo Braccini 2, Grugliasco, 10095, Italy; E-Mail: francesco.gai@ispa.cnr.it; 2 Department of Agricultural, Forest, and Food Sciences, University of Torino, Largo Braccini 2, Grugliasco, 10095, Italy; E-Mails: sonia.tassone@unito.it (S.T.); laura.gasco@unito.it (L.G.); 3 Agricultural Academy of Turin, Via Doria 10, Torino, 10100, Italy; E-Mail: giorgio_masoero@alice.it

**Keywords:** feces, rabbit, feed values, ruminant, biomethane potential, digestion, NIRS

## Abstract

**Simple Summary:**

This paper investigates the potential use of rabbit feces as a source of nutrition for ruminants and as an energy source. Feeding rabbit feces to ruminants or biogas production with rabbit manure may reduce the competition between human food and animal feed, provide a partial solution to some environment problems, and reduce treatment and disposal cost of manure. The *in vitro* rumen digestibility of rabbit feces was experimentally measured to assess its value as a feed for ruminants. In parallel, in order to extract the information about its potential nutritive and energetic value, the whole and partial relationships between the nutrient constituents of the rabbit feces and/or crop forages were investigated by chemometric analysis and also validated by near-infrared spectroscopy (NIRS). The results of this work indicated that rabbit feces has potential value as a ruminant feed and for biogas production.

**Abstract:**

There are prospects for using novel feeds from various sources to provide ruminants with alternative sources of protein and energy such as by-products, and animal wastes. Rabbit feces are a concentrated source of fiber and could have commercial potential both as input biomass in anaerobic processes for biogas production, as well as a fibrous source for ruminal degradation. The aims of this work were to assess the potential as ruminant feeding and as biogas production of rabbit feces, in comparison with 12 crops. The chemical composition and the potential and experimental *in vitro* true digestibility (IVTD) and neutral detergent fiber digestibility (NDFD) of 148 feces samples were determined by using chemical methods, Daisy system digestibility and/or NIRS predictions. The average biomethane potential (BMP) was 286 ± 10 l_CH4_/kg SV with −4% *vs.* the crops average. Milk forage unit (milk FU), IVTD and NDFD of feces were 0.54 ± 0.06 milk FU/kg DM, 74% ± 3% and 50% ± 5%, respectively, with comparisons of −19%, −11% and −24% *vs.* the crops average. Reconstruction of the potential values based on the chemical constituents but using the crop partial least square model well agreed with the NIRS calibrations and cross-validation. In a global NIRS calibration of the feces and crops the relative predicted deviation for IVTD, NDFD and milk FU were 3.1, 2.9 and 2.6, respectively, and only 1.5 for BMP. Running the Daisy system for rabbit feces in rumen fluid gave some inconsistencies, weakened the functional relationships, and appeared not to be correlated with the potential values of IVTD and NDFD. Nevertheless, the energetic potential of feces appears to be similar to some conventional crops at different degrees of maturity. Thus we conclude that rabbit feces has potential value as a ruminant feed and for biogas production.

## 1. Introduction

Livestock is currently one of the fastest growing agricultural subsectors in countries in agricultural development [1]. However, intensive farming will increasingly be affected by competition for natural resources, the need to operate in a carbon-constrained economy and particularly by competition between food and feed. There are prospects for using novel feeds from various sources to provide alternative sources of protein and energy such as by-products and animal wastes [1]. In the EU, the feeding of livestock manure is prohibited by Reg. (EC) No 767/2009 (Annex III), but it is a common practice throughout the world, used either directly or after transformation by chemical (acidification) or physical (heating) processes in feed formulations [2,3,4,5]. Animal manure is a rich source of lignocelluloses, polysaccharides, proteins, minerals and other biological materials [6,7]. Moreover, the accumulation of livestock excreta has been seen as an opportunity to recycle this material as biomass to produce biogas [8,9].

There is a significant move within many countries in agricultural development to increase the implementation of zero-grazing systems. This provides a timely incentive for biogas development in order to fully utilize the increased animal excreta captured at household level [10]. Several workers have studied the incorporation of different animal manures into various animal diets [2,5,11], but few studies have used rabbit feces [12,13], although they are higher in nutritional value than other animal wastes [14]. The results of the experiments indicate that manure can be successfully included in the feeds of both ruminants and non-ruminants, but animal waste is most efficiently used by ruminants such as cattle and sheep. Ruminants are able to digest fiber and to use non-protein nitrogen due to its microbial rumen population [15]. Moreover, in all these cases, the nutritional value of the manure was mainly a reflection of feed spillage, which is almost inevitable when intensive self-feeding systems are practiced. The high risk of disease arising from recycling livestock wastes, highlighted by the outbreaks of bovine spongiform encephalopathy, is now seen as a major deterrent to these practices [16].

Feeding rabbit feces for ruminants may reduce feed costs for smallholders, provide a partial solution to environment problems, and reduce the competition between food and feed in countries in agricultural development. In addition, rabbit feces are an abundant but under-used biomass resource for producing biobased chemicals and energy. This paper investigates the potential use of rabbit feces, accurately separated from urine with the aim to achieve a better handling and a better composition of the manure, as an add-on value for ruminant nutrition. The anaerobic microbiota of the rabbit gut and caecum is not useful for digesting plant walls, thus rabbit feces composition is characterized by a great abundance of neutral detergent fiber (NDF), nearly 60%. This fiber type is potentially degraded in a rumen by over 70%, thus providing an effective feeding value for ruminants. Great caution is needed for proper pasteurization of the feces. Processing of rabbit feces to be used as animal feed is necessary in order to destroy pathogens, improve handling and storage characteristics, and to maintain or enhance palatability.

The domestic rabbit has the potential to become one of the most important livestock species in countries in agricultural development due to its short generation interval, high fecundity and rapid growth rate [5]. Rabbits may be envisaged as a special case, because they are not involved in outbreaks between species: in fact, they need high feed fiber levels even though the feed fiber content is not completely digested neither by growing rabbits nor by rabbit does [17,18,19]. In light of these considerations, rabbit feces are concentrated in fibrous elements and could be considered interesting both as input biomass in anaerobic processes for biogas production, as well as a fibrous source for ruminal degradation.

The aims of the work were to assess the potential as ruminant feed and for biomethane production from rabbit feces, in comparison with 12 green crops from 8 botanic families. The rumen digestibility of rabbit feces was experimentally measured to assess its value as a feed for ruminants. In parallel, in order to extract the information about its potential nutritive and energetic value, the whole and partial relationships among the constituents of the rabbit feces and/or crop forages were investigated by chemometric analysis and also validated by near-infrared spectroscopy (NIRS).

## 2. Experimental Section

### 2.1. Data Sets and Analyses

The database, made up of 148 dried samples of feces, was derived from nine digestibility experiments with growing rabbits (Table 1) according to the harmonized methodology [20].

An aliquot of the sample feces was oven-dried at 90 °C for 24 h for dry matter (DM) determination, while the larger aliquot was dried in a forced-draft oven at 60 °C up to constant weight, air-equilibrated, ground in a Cyclotec mill and stored for later analysis. The dried samples were analyzed to determine the total N content according to the Dumas method, using a macro-N Nitrogen analyzer (Foss Heraeus Analysensysteme, Hanau, Germany), ash by ignition at 550 °C, NDF without sodium sulfite and α-amylase, and acid detergent fiber (ADF) as described by Van Soest *et al*. [21]. Gross energy (GE) was determined using an adiabatic calorimeter bomb (IKA C7000, Staufen, Germany).

*In vitro* true digestibility (IVTD) and *in vitro* NDF digestibility (NDFD) were analyzed by the Daisy II Incubator (Ankom, Tech. Co., Fairport, NY, USA) according to Robinson *et al*. [22]. The *in vitro* rumen incubations were performed in two consecutive fermentative runs. Ground samples (250 mg) were inserted into filter bags (Ankom F57 bags) which were then sealed. Digestion jars were filled with pre-warmed (39 °C) buffer solutions (266 mL of solution A: KH_2_PO_4_ 10 g/L, MgSO_4_ 7H_2_O 0.5 g/L, NaCl 0.5 g/L, CaCl_2_ 2H_2_O 0.1 g/L, Urea 0.5 g/L; 1330 mL of solution B: Na_2_CO_3_ 15.0 g/L, Na_2_S 9H_2_O 1.0 g/L) and placed into the incubator. Rumen liquor was collected from rumen contents obtained at a slaughterhouse and 400 mL of filtered liquor (through two layers of cheesecloth) was introduced into each jar together with the filter bags. After 48 h of incubation, the bags were removed, rinsed thoroughly with cold tap water and immediately analyzed for NDF content with the Ankom_200_ Fiber Analyzer and incinerated to correct the residual NDF for the residual ash.

**Table 1:** Data set characteristics of the 34 experimental iso-energetic and iso-nitrogenous diets involved in nine referenced trials.

Reference	Raw material	Diets	Feces
[23]	False flax ^a^	3	30
[24]	Chia ^b^	3	12
[25]	Golden flaxseed ^c^	3	12
[26]	Spirulina ^d^	4	12
[27]	DHEA/Spirulina/fat level ^e^	8	24
[28]	Perilla ^f^	3	12
[19]	Curcuma/oils ^g^	4	16
[29]	Dried tomato pomace ^h^	3	16
[30]	Live yeast ^i^	3	14

^a^ False flax (*Camelina sativa* L.) seed supplementation (0, 10% and 15%). ^b^ Chia (*Salvia hispanica* L.) seed supplementation (0, 10% and 15%). ^c^ Golden flaxseed (*Linum usitatissimum* L.) supplementation (0, 8% and 16%). ^d^ Spirulina (*Spirulina platensis*) supplementation (0, 5%, 10% and 15%). ^e^ Dehydroepiandrosterone (DHEA) supplementation (0 and 0.02%), or Spirulina (*Spirulina platensis*) supplementation (0 and 1%) and fat level (3% and 13%). ^f^ Perilla (*Perilla frutescens*) seed supplementation (0, 5% and 10%). ^g^ Curcuma (*Curcuma longa*) supplementation (0 and 0.3%) and oils (with 4% palm oil and 4% mais oil). ^h^ Dried tomato pomace supplementation (0, 3% and 6%). ^i^ Live yeast (*Saccharomyces cerevisiae* var. *boulardii*) supplementation (0, 0.03%, and 0.06%).

IVTD was calculated using the following equation:

(1) $$100\;-\;({\text{W}}_{3}\;-\;({\text{W}}_{1}\;\times \;{\text{C}}_{1}))\;\times \;100/({\text{W}}_{2}\;\times \;\text{DM})$$

where W_1_ is the filter bag weight, W_2_ is the sample weight, W_3_ is the final weight (filter bag + residue) after *in vitro* and sequential treatment with NDF solution, C_1_ is a comparison of the blank filter bag weight after and before digestion treatment and DM is the dry matter content of the samples.

NDFD was calculated using the following equation:

(2) $$100\;-\;({\text{W}}_{3}\;-\;({\text{W}}_{1}\;\times \;{\text{C}}_{1}))\;\times \;100/({\text{W}}_{2}\;\times \;\text{NDF})$$

where W_1_ is the filter bag weight, W_2_ is the sample weight, W_3_ is the final weight (filter bag + residue) after *in vitro* and sequential treatment with neutral detergent fiber solution, C_1_ is a comparison of the blank filter bag weight after and before digestion treatment and NDF is the neutral detergent fiber content of the sample.

Digestible and Indigestible NDF were calculated using the following equations:

(3) Digestible NDF = (NDF×NDFD/100)

(4) Indigestible NDF = NDF − Digestible NDF

### 2.2. Comparative Energetics

Energy value for ruminants was expressed as milk forage unit (Milk FU, according to Sauvant *et al*. [31]). Biomethane potential (BMP), expressed in l_CH4_/kg SV energetic value for biogas production, was assessed according to Thomsen *et al*. [32] by a mixture model:

(5) βC χC + βH χH + βL χL + βR χR + ε

where χC, χH, χL, and χR are the observed composition of the organic matter (OM) fraction of cellulose, hemicellulose, lignin and residuals (1-χC-χH-χL), respectively, and the error term ε represents uncertainty. Since the variables add up to one, an additional variable (χR) which is often called ‘residuals’ in relation to biomass composition, is included in the model. In this way, everything which is not carbohydrates or lignin is characterized as residuals,

(6) χR = 1 −(χC + χH + χL)

χR has not been considered in previous models as a regression variable, which might be problematic, since χR might contain methane yielding biomass constituents, such as lipids, fatty acids, pectin, proteins and tannins. The relationships were applied *in primis* to the 148 rabbit feces samples, and the results were also compared to a composite green forage dataset recently published by Tassone *et al*. [33].

### 2.3. Comparative Partial Least Square Models of Digestibility in Forages and in Rabbit Feces

The functional relationships between chemical composition and digestibility are studied within the previously cited data set [33] composed of 12 green forage crops sampled several times from 8 botanic families, namely (in increasing maturity type): *Boraginaceae* and *Chenopodiaceae*; *Lamiaceae*; *Asteraceae* and *Fabaceae*; *Cannabaceae*; *Brassicaceae*; *Linaceae*. In the present work, those functional equations found in forage crops, including both predictor variables and predicted variables (digestibility), are recalculated using a chemometric Partial Least Square (PLS) method (StatBox v.1.5, GrimmerSoft, Paris, France); the equations are then applied to the feces composition in order to predict the potential digestibility traits.

### 2.4. Calibration and Validation by NIRS

In this work, NIRS will first be used within the rabbit feces dataset to calibrate and cross-validate the energetic and nutritional properties, the Daisy system digestibility and the potential digestibility predicted using the crop PLS models. Second, a global calibration pooling the feces and the crop spectra was undertaken. The FT-NIR Spectrum IdentiCheck FT-NIR System (Perkin-Elmer, Beaconsfield, UK) was used to scan the rabbit feces samples in the extended NIR-MIR band from 714 nm to 3333 nm. The native spectra were imported into the WinISI II vers.1.5 software (Infrasoft International, Port Matilda, PA, USA) and elaborated after mathematical treatment 1,4,4,1. The spectra were fitted to predicted variables, namely energetic, experimental and potential digestibility of the feces; and statistics as a cross-validation and a Relative Prediction Deviation (RPD) were considered for performance evaluation. As a further step, a joint feces-forage dataset was explored in order to assess the relationships between them.

## 3. Results and Discussion

Table 2 reports the characteristics of the chemical composition of the feces and the compared crops. The rabbit feces showed less ash (−26%), ether extract (−15%), N-free extract (−16%) and residual fraction (−27%) contents but more crude protein (+18%) and GE (+10%) than the green crops. All the fiber components were boosted in feces, namely the NDF (+30%) and crude fiber (+39%) and also elevated for lignin (+42%) and ADF (+18%), showing the greatest increase in the hemicellulose content (+75%) and in the indigestible NDF part (+82%). With regard to the rabbit digestive physiology, it is well known that it needs moderate levels of functional fiber in feed, but the plant wall is only scarcely digested, both by growing rabbits and by does [17,18].

**Table 2:** Comparative composition and properties of the rabbit feces and the crops.

	Feces (F)			Ratio F/C	Crops (C)		
	Mean	SD	CV		Mean	SD	CV
*Composition*							
Gross Energy (MJ/kg DM)	17.85	1.76	0.10	10%	16.27	0.88	0.05
Ash (% DM)	10.37	2.58	0.25	−26%	13.95	3.61	0.26
Crude Protein (% DM)	15.50	1.91	0.12	18%	13.15	3.47	0.26
Ether Extract (% DM)	2.04	0.48	0.24	−15%	2.42	0.55	0.23
Crude Fiber (% DM)	32.44	1.96	0.06	39%	23.31	9.46	0.41
N-free Extract (% DM)	39.78	2.69	0.07	−16%	47.17	9.44	0.20
Residual Fraction (N)	0.40	0.05	0.11	−27%	0.55	0.06	0.11
NDF (% DM)	59.81	4.54	0.08	30%	45.93	7.35	0.16
Digestible NDF (% DM)	29.99	2.44	0.08	2%	29.53	3.99	0.14
Indigestible NDF (% DM)	29.82	5.10	0.17	82%	16.40	8.00	0.49
ADF (% DM)	39.59	2.22	0.06	18%	33.47	6.04	0.18
Hemicellulose (% DM)	20.22	4.23	0.21	75%	11.56	3.95	0.34
Cellulose (% DM)	28.99	2.28	0.08	11%	26.19	5.71	0.22
Lignin (% DM)	10.60	1.86	0.18	42%	7.45	1.57	0.21
Gross Energy (MJ/kg DM)	17.85	1.76	0.10	10%	16.27	0.88	0.05
*Properties*							
BMP (l_CH4_/kg SV)	286	10	0.04	−4%	297	8	0.03
Milk FU (/kg DM)	0.54	0.06	0.11	−19%	0.66	0.14	0.21

ADF, acid detergent fiber. NDF, neutral detergent fiber. BMP, biomethane potential. Milk FU, milk forage unit.

**Figure 1:**
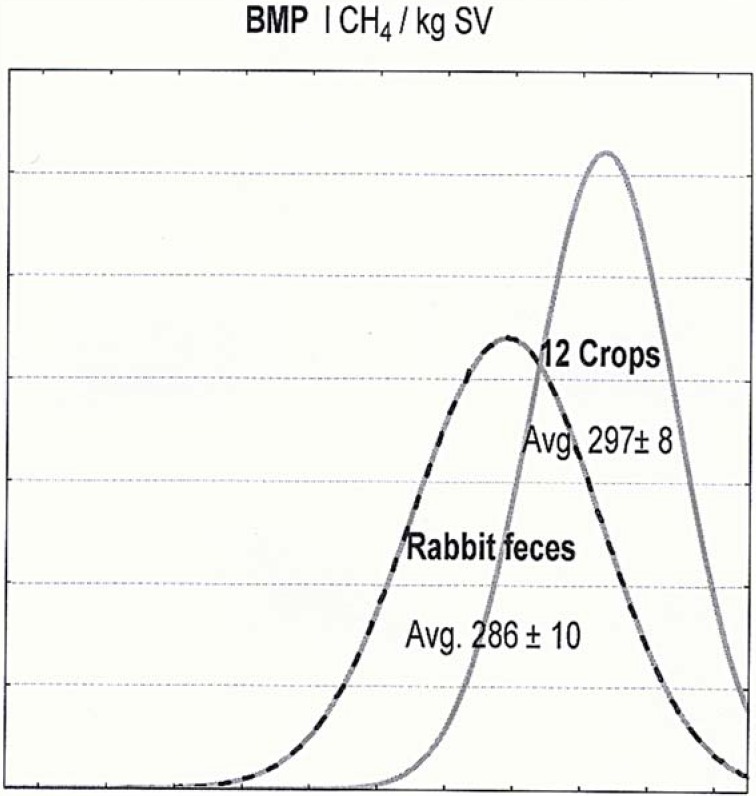
Distribution of the biomethane potential (BMP) of the crops compared to the rabbit feces.

Turning to its potential for anaerobic production of biomethane, the feces measured only 4% below the average of the 12 crops, and Figure 1 reports the respective neighboring distributions. By contrast, a great difference (–19%) penalized the estimated feeding value of the feces (avg. 0.54 ± 0.06 milk FU) as compared to the mean milk FU for the 12 crops (0.66 ± 0.14 milk FU).

**Figure 2:**
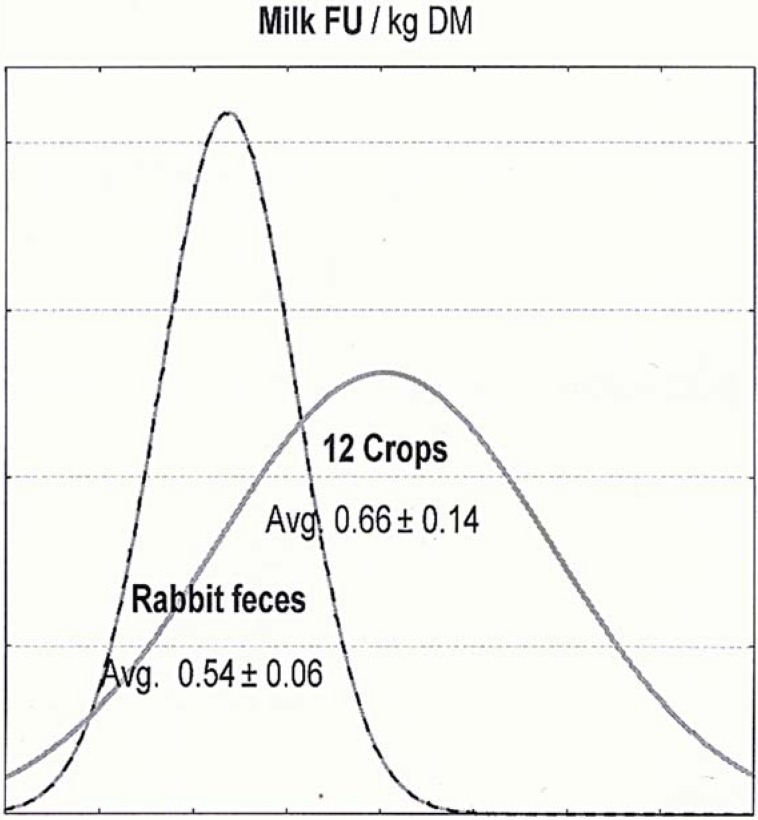
Distribution of the milk FU (Forage Unit) for the crops compared to the rabbit feces.

It is interesting to note the very different shape of the distributions in Figure 2: where the milk FU of the feces remained constant, the compared crops had most variability.

Table 3 reported the Partial Least Square (PLS) models for digestibility in rabbit feces and crops with standardized coefficients and statistics. Here, the situation appeared to be markedly different. In fact, in the crops, the fit of the Daisy system IVTD and NDFD was successful (R^2^ = 0.84 and 0.78), while in the feces the fit was fair (R^2^ = 0.23 and 0.10) for both parameters. No references in literature about such *in vitro* Daisy system applications on feces are available.

Thus the origin of this default can be ascribed to several causes: interference by the two microbiota, because the feces were dried at 60 °C but not sterilized; a complex transformation of the indigestible NDF fraction occurred in the rabbit gut; there was an overabundant presence of hemicellulose in the rabbit feces.

**Table 3:** Partial Least Square (PLS) models for digestibility in rabbit feces and crops with standardized coefficients and statistics.

	IVTD	IVTD	NDFD	NDFD
	Feces	Crops	Feces	Crops
Ash (% DM)	−0.081	0.031	−0.042	0.063
Gross Energy (MJ/kg DM)	−0.048	−0.030	−0.131	−0.053
Crude protein (% DM)	0.108	0.152	0.091	0.130
Ether Extract (% DM)	0.107	0.088	0.086	0.083
Crude fiber (% DM)	−0.041	−0.134	−0.069	−0.117
N-free extract (% DM)	0.027	0.062	0.025	0.040
ADF (% OM)	−0.133	−0.158	−0.163	−0.149
NDF (% OM)	−0.223	−0.214	−0.231	−0.180
Lignin (% OM)	0.078	0.024	0.043	−0.004
Hemicellulose (% OM)	−0.169	−0.048	−0.163	−0.032
Cellulose (% OM)	−0.194	−0.076	−0.193	−0.093
XResidual (N)	0.223	0.098	0.231	0.110
BMP (l_CH4_/kg SV)	−0.129	−0.070	−0.095	−0.046
Milk FU (/kg DM)	0.180	0.182	0.194	0.165
R^2^	0.23	0.84	0.10	0.78
Standard Error of Calibration	7.50	2.98	8.45	5.97
Mean of experimental	66.9	83.2	51.1	65.8
SD of experimental	8.5	7.4	8.9	12.6
Mean of potential in feces	74.4		50.2	
SD of potential in feces	3.0		4.5	
*r*	0.85		0.89	

IVTD, *in vitro* true digestibility. NDFD, neutral detergent fiber digestibility. ADF, acid detergent fiber. NDF, neutral detergent fiber. Milk FU, milk forage unit. BMP, biomethane potential. R^2^ = r-square. SD = Standard deviation. *r =* Correlation of the standardized PLS coefficients.

Nevertheless, when looking at the functional relationships, calculated in terms of standardized PLS coefficients, the feces appeared very similar to the crops because the correlation between the two coefficients was 0.85 for IVTD and 0.89 for NDFD, thus the lack of fit derives from a very great noise of the single fecal specimens when put in the Daisy system. On the strength of this fact, which means that crops and feces matrices in the Daisy system have similar features, the potential IVTD and NDFD were reconstructed for the feces using the equation established in the crops. The reconstruction led IVTD to increase to a potential level of 74.4%, a value which is only 11% lower than the 83.2% recovered in the crops, while the NDFD did not change the mean of the experimental Daisy system value, but greatly elevated the fit of the system.

The NIRS cross-validation performances for the experimental Daisy system values were fair (Table 4): indeed, the 1-VR coefficients were 0.41 and 0.36 for the feces IVTD and NDFD. After the experimental results were substituted by their potential values, the fit increased to 0.67 and 0.70, corresponding to RPD values of 1.7 and 1.8. When the global feces-crops calibration was attempted, the fit of the Daisy system digestibility parameters, real for the crops and potential for the feces, further increased to an RPD of 3.2 and 2.9, respectively, a level regarded as safe for applications. The milk FU fit for the feces was already just as high: an RPD value of 1.9 concerned the NIRS spectra of the feces, increasing to 2.6 by adding the crops.

**Table 4:** Near-infrared spectroscopy (NIRS) calibration and cross-validation of the energetic and nutritional properties of the rabbit feces and of the crops separately by feces and pooled with crops.

Variable	NIRS Spectra	Method	Mean	SD	RSQ	SECV	1-VR	RPD
BMP	Feces	Thomsen *et al*. [32]	288.6	6.01	0.53	5.28	0.23	1.1
	Feces and Crops		292.2	7.16	0.87	4.63	0.58	1.5
Milk FU	Feces	Sauvant *et al*. [31]	0.5	0.06	0.98	0.03	0.72	1.9
	Feces and Crops		0.6	0.10	0.94	0.04	0.85	2.6
IVTD	Feces	Daisy	71.6	6.37	0.86	4.90	0.41	1.3
	Feces	Potential	74.5	2.88	0.91	1.65	0.67	1.7
	Feces and Crops	Potential	77.4	6.39	0.94	2.03	0.90	3.2
NDFD	Feces	Daisy	50.1	10.36	0.64	8.32	0.36	1.2
	Feces	Potential	50.5	4.44	0.92	2.43	0.70	1.8
	Feces and Crops	Potential	55.2	10.33	0.93	3.52	0.88	2.9

BMP, biomethane potential. Milk FU, milk forage unit. IVTD, *in vitro* true digestibility. NDFD, neutral detergent fiber digestibility. Mean = mean of the constituents in the whole dataset; SD = Standard deviation of the whole dataset; RSQ = r-square in calibration mode; SECV = standard error in cross-validation mode; 1-VR = 1-variance ratio; RPD = relative prediction deviation (=SD/SECV).

In contrast, the biomethane energy values appeared unpredictable by the NIRS of the feces (RPD = 1.1) and also by the combined dataset (1-VR = 0.58; RPD = 1.5). The estimates of BMP predicted by the Thomsen et al. [32] methods are apparently not structured in the NIRS radiation of the feces. When the spectra of the feces were integrated with that of the crops, the dataset was more similar to the meadow grasses reported by Raju *et al*. [34], but the fit of *in vitro* organic matter Digestibility was below their reported values 1-VR = 0.69 with RPD = 1.75.

## 4. Conclusions

Using rabbit feces as feedstuffs for ruminants is conceptually attractive. The energetic potential of feces appear to be similar to some conventional crops at different degrees of maturity, thus this material could be included in biomethane digesters; however, effective trials should focus on possible boomerang effects arising from the presence of active microbes or of anti-microbial substances. Running the Daisy system for feces in rumen fluid gave some inconsistencies, weakened the functional relationships, and appeared not to be correlated with the potential values of IVTD and NDFD.
